# Nutrient-Limited Operational Strategies for the Microbial Production of Biochemicals

**DOI:** 10.3390/microorganisms10112226

**Published:** 2022-11-10

**Authors:** Hemshikha Rajpurohit, Mark A. Eiteman

**Affiliations:** School of Chemical, Materials and Biomedical Engineering, University of Georgia, Athens, GA 30602, USA

**Keywords:** chemostat, *Escherichia coli*, fed-batch, metabolic engineering, nutrient-limitation, *Saccharomyces cerevisiae*

## Abstract

Limiting an essential nutrient has a profound impact on microbial growth. The notion of growth under limited conditions was first described using simple Monod kinetics proposed in the 1940s. Different operational modes (chemostat, fed-batch processes) were soon developed to address questions related to microbial physiology and cell maintenance and to enhance product formation. With more recent developments of metabolic engineering and systems biology, as well as high-throughput approaches, the focus of current engineers and applied microbiologists has shifted from these fundamental biochemical processes. This review draws attention again to nutrient-limited processes. Indeed, the sophisticated gene editing tools not available to pioneers offer the prospect of metabolic engineering strategies which leverage nutrient limited processes. Thus, nutrient- limited processes continue to be very relevant to generate microbially derived biochemicals.

## 1. Introduction

Biochemical processes use enzymes or microorganisms as catalysts to convert a substrate to a product, and are analogous to chemical processes in terms of basic design parameters including stoichiometry, energy relationships, kinetics, and equilibria [1]. Design and implementation of microbial bioprocesses additionally require an understanding of metabolic activity and its effect on growth and product formation as well as factors which influence organisms and these cellular reactions.

**Figure 1:**
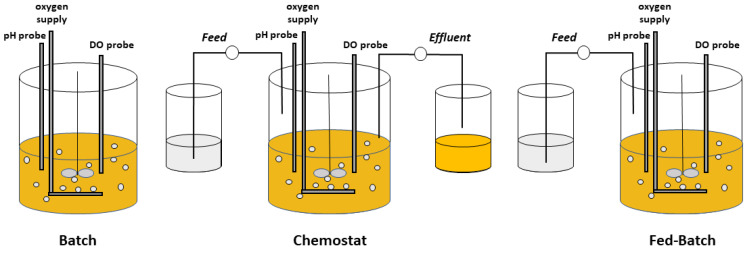
The three general modes of fermentation operation: batch, continuous, and fed-batch processes.

Operationally, microbial bioprocesses are often classified by the strategies used for substrate addition and product removal (Figure 1). In a *batch* process, the carbon-substrate/energy source as well as other nutrients are at the onset charged into the reactor, and product is harvested at the conclusion of the process, typically when the carbon source is depleted. Although material can be introduced into the reactor for oxygenation, pH control, foaming, and other minor reasons, a batch process is considered a closed system. Other operations in contrast are implemented as distinctly open systems. A *chemostat* occurs when the flowrates of a nutrient-containing feed and an effluent of products and residual nutrients are equal [2]. The dilution rate (*D*) normalizes that feed rate *F* by the constant volume of the system *V* (*D* = *F*/*V*). In addition, any of several semi-continuous *fed-batch* processes involve the feeding of nutrients often without product withdrawal [3,4]. Variations on these basic operational modes include a *turbidostat* [5,6], wherein the feed rate is controlled to maintain a constant cell density, and an *accelerostat* [7], wherein the dilution rate is changed progressively and slowly to maintain prolonged pseudo-steady-state conditions over a range of growth rates. A two-reactor cascade in which biomass formation and product formation are spatially distinct is another novel continuous operational mode which increases volumetric productivity of intracellular products [8]. Several of the methods for continuous cultivation have been critically compared for their utility in high-resolution characterization of metabolism [9].

Microbial bioprocesses are further categorized by whether or not the growth of the microorganisms is *limited* by the availability of nutrients. The term descriptively suggests a process in which cellular growth and metabolism are less than maximal as a result of the limited availability of one or more nutrients during the course of the process. Whereas many authors use *limitation* in the context of cessation of growth (e.g., [10]), we use the term to refer to the state in which cells are indeed metabolizing nutrients and even steadily growing, albeit at a lower than maximal rate because of the *limited* availability of one or more nutrients, a condition which can be sustained simply by feeding that limiting nutrient at a controlled rate. A batch process is not a nutrient-limited process (at least until the very end of the process), whereas a chemostat must be a nutrient-limited process. A fed-batch process might or might not be nutrient-limited, depending on the nature of the feeding. Fed-batch operations may also be distinguished by whether or not feedback control is used in establishing the feed [11], a classification which focuses on externally applied control rather than on the resulting physiological behavior of the cells. The type of nutrient limitation has a profound effect on the performance of cells in a bioreactor. This review does not consider control algorithms and strategies, for example, in the context of protein production [12].

In order to establish whether or not cell growth is nutrient-limited, one must consider that a relationship exists between the concentration of any nutrient and the specific growth rate. Among the many models available, the relationship is often satisfactorily described by the hyperbolic Monod Equation [13,14]:

(1) $$\mu =\mu_{max}\left(\frac{S}{K_{s}+S}\right)$$

where *µ_max_* is the maximum specific growth rate (h^−1^) which occurs in the absence of inhibitors and when nutrients are plentiful under those culture conditions, *K_S_* is the saturation constant (e.g., g/L), and *S* is the nutrient concentration (g/L). Although a great simplification of true microbial growth dynamics, the Monod Equation does capture the idea that a single nutrient can limit the specific growth rate, and shows that the growth rate approaches the maximum specific growth rate when *S* >> *K_S_*. The saturation “constant” is not actually a constant, but itself depends on culture conditions (e.g., temperature, pH), the nutrients themselves (e.g., glucose versus glycerol; ammonium versus nitrate), and even the degree of adaptation of the cells [15]. The value of *K_S_* reflects the overall metabolic affinity for a substrate and can be affected by altering the enzyme kinetics of a single reaction in cellular metabolism [16]. Considering the example of *Escherichia coli* with glucose as the carbon source, values for *K_S_* have been reported as low as 50 μg/L [15,17] to greater than 8 mg/L [18,19,20]. Regardless of the precise numeric value of *K_S_*, these results demonstrate that the saturation constant can be three orders of magnitude lower than a typical initial substrate concentration, so that the specific growth rate is essentially independent of *S* for the duration of a batch process. Since the growth rate is usually less than maximal only at very low nutrient concentrations, using a sensor feedback control strategy directly to maintain the concentration of a carbon/energy source or another nutrient at limiting conditions is generally impractical. Despite the difficulty in determining a value for the saturation constant [21], the Monod Equation is useful. For example, the equation can also be used to estimate the expected concentration of the limiting nutrient (i.e., when the process is operated in that way). Specifically, by rearranging the Monod Equation, the concentration of the growth-limiting nutrient (*S_lim_*) needed to sustain a given growth rate (*μ* < *μ_max_*) is:

(2) $$S_{lim}=\frac{\mu K_{s}}{\mu_{max}-\mu }$$

Due to their simplicity, batch processes are often used to study many microbial phenomena such as growth, carbohydrate utilization, and product formation. However, the flexibility and degrees of control in a batch process are limited. Because the microbes in a batch process invariably encounter nutrients in excess relative to *K_S_*, the microbes grow for essentially the entire process at their maximum rate after an initial acclimatization, or lag period, until one or more nutrients suddenly become depleted. Thus, one has no operational ability to influence the nutrient-dependent physiological state of the culture. Nutrient-limited processes are underappreciated, particularly in the context of modern molecular tools, and a goal of this review is to highlight current knowledge on these processes.

## 2. Chemostat Processes

Originally developed to study bacterial cultures for a long duration [22], the chemostat maintains the cell growth rate lower than the maximum specific growth rate (*μ_max_*). The chemostat is an indispensable tool to investigate how growth rate affects cellular processes and how cells or consortia of cells evolve in response to nutrient limitation as a selective pressure [23], though it is particularly difficult to operate for microbes which grow on walls or aggregate such as filamentous fungi [24]. One can dictate which nutrient limits cell growth (e.g., C, N, P, etc.) merely by adjusting the medium composition [25]. Mean residence time in the chemostat is equal to the inverse of the dilution rate 1/*D* [26]. If the selected dilution rate is lower than the maximum specific growth rate (*μ_max_*) at the given environmental conditions, then a steady-state is normally achieved: the growth rate the microbes experience (*μ*) becomes equal to *D*, and the limiting nutrient attains a low, constant concentration (e.g., Equation (2)). If the dilution rate approaches or exceeds the maximum specific growth rate, then the cells cannot grow as quickly as the medium is withdrawn, leading to net loss of cells and culture washout. Because chemostat processes maintain a high biomass concentration compared to batch processes, a chemostat achieves a fairly high product and biomass volumetric productivity or space-time yield. Unfortunately, a continuous operation has an increased prospect of contamination or genetic drift during a prolonged biological process because of competition for the limiting nutrients [27,28,29]. Indeed, given the likelihood for mutations, a chemostat does not strictly reach a physiological “steady-state” [30]. In fact, at a long time-scale, the chemostat is an invaluable tool to evolve strains with greater substrate affinity [31,32,33,34], with relieved auxotrophy [35] or with a gain of substrate utilization [36]. Different limiting nutrients (e.g., N-limited versus Fe-limited) change the profile of mutations that occur, and the rate of mutations [37]. Nutrient-limited conditions also encourage phenotypic heterogeneity [38].

Care must be made in medium design for nutrient-limited processes because cells use a portion of the carbon/energy source for maintenance and also often to accumulate storage products. Therefore, the proportion of nutrients needed for a growth limitation itself depends on the growth rate, and dual-nutrient-limited regions are possible [39,40,41,42]. One fixed medium composition can lead to C-limited growth at a low dilution rate, dual C/N-limited growth at an intermediate dilution rate, and N-limited growth at a high dilution rate [40]. Nevertheless, operation of a chemostat is useful to identify optimal conditions for fed-batch operations for biochemical generation (e.g., [43]), and therefore, the chemostat is an indispensable tool for bioprocess development. Chemostat processes have been previously reviewed in the context of recombinant protein production [44], in which case C-limited growth is typically employed to avoid overflow metabolism, a situation in which extra carbon is diverted to an undesirable by-product such as acetate [45]. This review focuses on processes which are limited by another element and are thus carbon-excess, and the effect of these processes on microbial physiology.

## 3. Fed-Batch Processes

Fed-batch is a semi-continuous process in which one or more nutrients are supplied to the growing microbial culture with or without periodic withdrawal [11]. A fed-batch process can be implemented so that nutrients are intermittently added to the reactor and are always in excess from the perspective of the cells (i.e., relative to *K_S_*), resulting in a process physiologically similar to a batch process. Such a culture allows prolonged growth at *µ_max_* and can be considered a non-limited-nutrient fed-batch or a *repeated fed-batch*, although the latter term is often applied to a fill-and-draw process in which a portion of culture is periodically withdrawn and fresh nutrients supplied [46]. This type of fed-batch process is particularly beneficial in cases for which a high concentration of a particular nutrient inhibits microbial growth, since that nutrient can be maintained below its inhibitory concentration while achieving a near maximal growth rate [47,48]. Alternatively, a *nutrient-starved fed-batch* is a common process which involves growing a culture first with ample nutrients, and then in a subsequent cycle, supplying the culture with a feed in which at least one essential nutrient is absent. In such a nutrient-starved process, often the carbon/energy source is supplied to satisfy the cultures’ maintenance requirement, while the *absence* of another nutrient (e.g., N) prevents growth [49]. In this case, cells cannot continue growing without the missing essential nutrient(s), but they often remain able to metabolize the supplied carbon source and accumulate storage products or transform that carbon source into a desired product [50,51,52,53].

A fed-batch process can also be implemented as a *nutrient-limited fed-batch*, which like a chemostat necessitates that a nutrient feed is introduced at a rate lower than the growing culture can maximally metabolize. In contrast to a nutrient-starved process, though, the culture is growing continuously, and the rate of introducing the limiting nutrient controls the metabolic and growth rates [54]. Because no effluent typically exists, cells and products remain in the culture, enabling fed-batch processes to achieve high cell density and product concentration. A quasi-steady state is achieved in nutrient-limited fed-batch cultures [54].

Nutrient-limited fed-batch processes can be operated in several ways. For example, the growth-limiting nutrient can be introduced into the culture at a constant rate or at an exponential rate [11]. When a growth-limiting nutrient is fed at a fixed rate (*F_c_*), the growth rate of the culture decreases with time [11,55]. In an exponential fed-batch process the nutrients are fed at an exponentially increasing rate to match the needs of the growing culture, to maintain a constant growth rate *μ_c_* less than *μ_max_*. The time-varying feed rate of a process limited by the carbon-energy source during an exponential fed-batch process is:

(3) $$F=(V_{i}X_{i}/S_{f})\left(m_{s}+\frac{\mu_{c}}{Y_{X/S}}\right)e^{\mu_{c}t}$$

where volumetric feed rate (*F*) is related to initial cell concentration (*X_i_*), initial volume (*V_i_*), substrate concentration in feed (*S_f_*), biomass yield coefficient (*Y_X_*_/*S*_), cell maintenance coefficient (*m_s_*), and the desired constant specific growth rate (*µ_c_*) [56]. An exponential fed-batch operational mode has been used to obtain high cell density, avoid oxygen limitation, minimize metabolic heat generation, and minimize by-product formation [56,57,58]. Like the saturation constant, the maintenance coefficient should not be considered a constant value, as it includes multiple cellular phenomena that may change over the course of a process [59,60]. This simple parameter is merely an attempt to encapsulate quantitatively the portion of substrate consumption not used for growth or for product formation.

## 4. Nutrient Limitation Compared to Nutrient Starvation

From the cells’ perspective, nutrient *limitation* is quite different from nutrient *starvation* [61], the latter which is often called a “resting cell” process. Nutrient limitation permits continued, possibly steady-state growth, whereas starvation in one or more essential nutrient induces dynamic stress responses and ultimately prevents further growth [62,63,64]. Many researchers examine the effect of nutrient scarcity by *restricting* an essential nutrient in the medium to cause that nutrient to be depleted first, or by transferring washed and concentrated cells from a complete to a depleted medium (e.g., [65]). Similarly, batch processes are typically composed of a medium from which carbon is depleted first, leading many to refer to such a batch process as “carbon-limited”. The term “starved” would be preferred to describe a batch process: only after a period of maximal growth do the cells transition quickly to non-growth due to first deficiency then absence of that one (or more) nutrient. The distinction is more important when a nutrient other than the carbon source is the first to be depleted, because the remaining excess carbon/energy source often continues to be consumed and converted into an intracellular or extracellular product despite, or often because of, the lack of growth.

Because of these physiological differences, care should be taken when comparing results from nutrient *starvation* with nutrient *limitation*, wherein growth is maintained by the slow addition of one or more limiting nutrients. The literature is replete with studies describing “nutrient limitation”, when in fact the initial medium was merely adjusted so that the cells experience a short batch process until a specified nutrient is depleted, that is, the cells transition from a higher growth rate to being *starved* for that nutrient. Typically, these studies examine the culture for the metabolism of a remaining excess carbon source while the culture transitions from nutrient scarcity to nutrient exhaustion. *Nutrient limitation* implies a process in which the cells *continue to grow*.

To understand the physiological response of an organism to nutrient limitation, it is very important to have controlled conditions (pH, temperature, and oxygen) and a well-defined medium since common complex components such as yeast extract and peptone complicate the identification of the limiting nutrient and interpretation of the cellular response [26,66]. For example, enzymes in a pathway to a particular required metabolite (e.g., a vitamin) may require iron, and if the complex medium contains a small quantity of that metabolite, then the effect of iron limitation could differ in that complex medium compared to a medium with a single carbon/energy source. Comparing vastly different media compositions, using complex medium components such as protein hydrolysate, or using microbial consortia can make identification of the limiting nutrient(s) virtually impossible (e.g., [67,68,69]), and make results difficult to interpret.

One important measurement associated with nutrient limitation and cell growth in general is the biomass yield (Y, Table 1), which expresses the quantity of cells on a dry basis generated per quantity of nutrient consumed (e.g., units of g cells/g nutrient). For nutrients not used as an energy source or in a product, the biomass yield is the reciprocal of fractional composition of that nutrient (e.g., g nutrient/g cell). The steady-state biomass yield of a nutrient is greatest when that nutrient is limiting. For example, the *E. coli* biomass yield on nitrogen (Y_X/N_, dry basis) is 8.8–9.8 g cells/g N during an N-limited chemostat, but 7.8–7.9 g cells/g N during glucose-limited growth, and 7.2–7.5 g cells/g N during Fe-limited growth [70]. In other words, *E. coli* is composed of 10.1–11.4% N when N is limiting, but 12.5–13.5% N when N is in excess and the cells are limited by something else: a loss of about 20 mg of N in one gram of cells when growth is limited by N. Similarly, *E. coli* biomass yield on iron (Y_X/Fe_) is about 100,000–150,000 g/g (i.e., 8 μg Fe per g of cells) during an Fe-limited chemostat [71], but is 5000–7000 g/g (200 μg Fe per g of cells) in Fe-excess batch cultivation [72]. These bacterial cells are thus composed of roughly 20–25 times more Fe when excess Fe is in the medium compared to when this element is limiting (largely irrespective of growth rate). These observations demonstrate how the physiological steady-state differs with conditions, and how cells would likely undergo an extraordinary dynamic response in an Fe-starvation process. Cells initially experiencing low but excess Fe would transition through a wide range of states (from 200 μg Fe to 8 μg Fe per g of cells) before becoming truly depleted in Fe and unable to grow further. Such a dynamic response has been noted with high resolution in nitrogen-limited anaerobic processes using *Saccharomyces cerevisiae*, where a 7% increase in biomass occurs shortly after N-depletion, largely as a consequence of trehalose and glycogen accumulation [73]. Such a growth dynamic cannot be explained exclusively by the Monod equation, and requires the use of at least two independent growth rates, one representing growth in the presence of N, and a second corresponding to carbohydrate-accumulating growth after N-depletion [74]. The unsurprising lesson is that cells have great flexibility to adjust their composition in response to the environment [40], and usually minimize their composition of a limiting nutrient at the expense of energy efficiency, typically by upregulating pathways using less of that nutrient, or otherwise avoiding unnecessary use of the limiting nutrient. As cells optimize their metabolic network, biomass yields also vary with growth rate and temperature [75].

The method of measurement affects the calculation of the yield coefficient. One method is called a “pulse shift technique” [76,83]. This technique involves achieving a steady-state, and then injecting a pulse of a suspected growth-limiting nutrient into the reactor. If the injected nutrient is indeed growth-limiting, then the culture will no longer be limited by that nutrient, and the biomass will increase until the injected nutrient is depleted. Typically, an elemental analysis is not performed on the medium, the technique does not account for potentially multiple, simultaneous nutrient limitations, and it does not consider the cellular flexibility that an injected nutrient might be incorporated into cells without an observed biomass change. Thus, values calculated using this technique are generally low compared to values using other measurements. The dry mass of the cells is measured by drying a selected volume of washed cells at defined conditions of temperature and duration, and thus, this measurement also introduces variability into the yield calculation.

Most biochemical products of interest are composed of carbon, oxygen, and hydrogen. A common approach to maximize microbial formation of such products is by a two-step culture system in which a first phase achieves high cell density potentially without nutrient limitation, followed by a second phase, in which cells are either limited (allowing some growth) or starved (allowing no growth) by the absence of one or more nutrients other than C (e.g., [90,91,92]). After achieving a high biomass concentration, such nutrient starvation/limitation generally maximizes carbon conversion to the product at the expense of additional biomass. For example, intracellular storage products such as poly(hydroxybutyrate) accumulate under N-, P-, S-, or Mg-limitation [93,94,95]. In an analogous fashion, photosynthetic microbes [96,97,98] and plants [99] exude carbon under non-carbon nutrient limitation. The scope of this review is to consider the physiological effects of microbes growing under conditions of limitation or starvation by nutrients other than carbon.

## 5. Physiological Effects of Non-Carbon Nutrient Limitation

### 5.1. Nitrogen

Nitrogen occurs throughout cells, in proteins, nucleotides, and many metabolites. Nitrogen as ammonium is assimilated in *E. coli* and most bacteria and yeast into glutamine and glutamate [100], which are the primary intracellular nitrogen donors. In bacteria, 88% of the cellular nitrogen is derived from glutamate, while 12% is derived from glutamine [101]. Glutamate is the most abundant metabolite in *E. coli*, accounting for about 40% of the total metabolite concentration [102]. In many bacteria such as *E. coli*, two ammonium-assimilating pathways are available, a NADPH-dependent glutamate dehydrogenase and a high-affinity glutamate synthase (glutamine oxoglutarate aminotransferase, GOGAT)/glutamine synthase. Glutamate dehydrogenases generally have high values of K_M_ for ammonium, so that during N-limited growth, glutamine synthase expression is elevated to maintain sufficient glutamate [103]. Dynamic N starvation in *E. coli* and *S. cerevisiae* growing on glucose depletes glutamine and to a lesser extent glutamate while α-ketoglutarate increases markedly and can even be excreted [65,104]. Accumulation of α-ketoglutarate occurs in cyanobacteria also, a signal which upregulates nitrogen assimilation via the global regulator NtcA and other regulators [105,106]. In yeast, the concentration of tryptophan, which relies on glutamine for its synthesis, decreases, while phenylalanine and tyrosine, which rely on glutamate for nitrogen, do not change, resulting in an accumulation of phenylpyruvate and phenylethanol, a quorum-sensing signal [107]. Accumulated α-ketoglutarate in *E. coli* noncompetitively and cooperatively inhibits EI of the PTS [108], citrate synthase [109], and PEP synthetase [110]. Furthermore, sudden nitrogen availability in N-starved wild-type *E. coli* induces a decrease in α-ketoglutarate and rapid increase in glucose uptake rate, while in a PTS-deficient strain with elevated galactose permease, glucose uptake is insensitive to N-availability [108]. In general, *S. cerevisiae* N-limitation leads to depletion of intracellular amino acids, particularly at low dilution rates [104]. Because intracellular glutamine and arginine concentration correlates strongly with dilution rate, these compounds likely control growth in N-limited *S. cerevisiae* [104]. *Synechocystis* also accumulate α-ketoglutarate [111] under N-starvation as well as glycogen, which is associated with the induction of the *glgX* gene [112,113,114]. *E. coli* has a high protein turnover under N-limited conditions compared to C-limited or P-limited conditions [115].

Under steady-state conditions, cells tend to have greater uptake of the carbon/energy source when grown under carbon-excess conditions compared to carbon-limited conditions. For example, under N-limited conditions at 0.2 h^−1^, *E. coli* shows a 2.3-fold greater specific glucose-consumption compared to under glucose-limited conditions [116], while *S. cerevisiae* shows 2–3 greater specific glucose consumption compared to glucose-limited conditions at all growth rates [117]. This extra carbon at the same growth rate is diverted to energy consuming reactions, and *E. coli* also generates substantially more acetate under N-limited conditions compared to C-limited conditions: 0.19 g/g during N-limited growth at 0.10 h^−1^, but no acetate during C-limited growth [118]. In *E. coli*, several genes associated with TCA cycle enzymes (isocitrate lyase, fumarase, succinate dehydrogenase) are downregulated under N-limitation compared to glucose-limitation, while genes of the Embden–Meyerhof–Parnas and pentose phosphate pathways are induced [119]. N-limited conditions generally increase the flux through glycolysis relative to the pentose phosphate pathway [116]. Some cells grown under carbon excess conditions induce ATP-dissipating futile cycles. For example, under N-limited conditions, *Bacillus subtilis* increases flux through the oxaloacetate-PEP-pyruvate cycle (PEP carboxykinase, pyruvate kinase, pyruvate carboxylase) leading to the net loss of one ATP per cycle [77]. N-limited *S. cerevisiae* cultures showed much lower intracellular concentrations of NAD and NADH compared to C-limited cultures [117]. Increasing dilution rate of *S. cerevisiae* increased the CO_2_ generation under N-limited conditions. In general, CO_2_ emission increases with growth rate irrespective of nutrient limitation [77,119], though CO_2_ evolution is greater under N-limitation compared to other nutrient limitation.

N-limitation, or more generally C-excess conditions, favor the formation of many biochemical products. For example, citric acid generation by *Aspergillus niger* is typically carried out under N-limited or dual N-/P-limited conditions [120,121], which are preferred operational modes because of nitrogen catabolite repression [122]. *Candida oleophila* also generates 0.7 g/g citrate under N-limited conditions at a dilution rate of 0.0185 h^−1^ [123], while *Penicillium simplicissium* excreted both malate and citrate under N-limited conditions [124], and *Yarrowia lipolytica* generated nearly 90 g/L citrate at the lowest dilution rate (<0.01 h^−1^) though the greatest yield of 0.67 g/g was at a greater dilution rate [125]. Citrate formation by *Penicillium ochrochloron* under N-limited (and P-limited) conditions is correlated with much lower nucleotide concentrations [126]. Similarly, N-limitation yielded the largest quantity of lipids in an oleaginous *Candida* [127], *Cryptococcus curvatus* [128], and *Y. lipolytica* [129]. In *Schizochytrium* sp., ammonium starvation resulted in the greatest squalene content (223 mg/g of total lipids) compared to phosphate starvation (30 mg/g) or excess nutrients (143 mg/g) [130]. N-limited chemostats at 0.1 h^−1^ resulted in 59% of the catabolic flux directed to 1,3-propanediol, whereas under P-limited conditions, 43% of the flux was directed to this product [131]. In N-limited fed-batch cultures, over 60 g/L 1,3-propanediol was obtained with a productivity of 1.7 g/L·h. Nitrogen starvation increased itaconate production ten-fold compared to batch conditions in a *Corynebacterium glutamicum* strain expressing cis-aconitate decarboxylase from *Aspergillus terreus* [132]. Very low growth rate of recombinant *S. cerevisiae* under N-limited conditions generated over 0.61 mol/mol succinate for 500 h [133]. A two-reactor N-limited chemostat system, allowing very low dilution rates, yielded about 200 g/L erythritol using *Y. lipolytica* with a yield of 0.66 g/g glycerol [134,135].

Storage products such as polyhydroxyalkanoates are well-known to accumulate under N-starvation (the processes are typically referred to as N-limitation). One approach is to grow *Alcaligenes eutrophus* (*Cupriavidus necator*) to a high cell density, and then stop providing N while maintaining a high glucose concentration [93,94,136]. Such N-starvation led to about 120 g/L poly(hydroxybutyrate) or poly(3-hydroxybutyrate-co-3-hydroxyvalerate), depending on co-substrates provided. A similar approach using *Alcaligenes latus* leads to 112 g/L poly(hydroxybutyrate) with a productivity of about 5 g/L·h from sucrose [137]. The cyanobacteria *Synechocystis* also accumulate poly(hydroxybutyrate) during N-starvation [138,139], an observation attributed to the induction of the *phaC* gene coding PHA synthase during N-starvation [140].

Compared to C-, S- or P-limited conditions, *S. cerevisiae* generates the greatest ethanol yield (0.35 g/g) at 0.1 h^−1^ under N-limited steady-state conditions [141]. N-starvation during the production of actinorhodin by *Streptomyces lividans* also led to the formation of α-ketoglutarate [142]. N-starvation (referred to as limitation) led to the accumulation of cellobiose lipids in two ustilaginomycetous yeasts [143].

Although *Clostridium acetobutylicum* under C-limited conditions at neutral pH leads to acetate and butyrate formation [144], N-limited conditions at a pH below 5.2 result in acetone and butanol solvent formation, but at a decreased rate [145]. No solvent formation is observed under N-limited conditions at pH 5.7 [146]. Quite surprisingly, N-limited conditions result in the accumulation of pyruvate and amino acids such as valine in *C. thermocellum*, a result attributed to a shift in pyruvate-ferredoxin oxidoreductase and an increased malic enzyme flux [147].

N-limited conditions are widely used and easily implemented. N-limitation increases glycolytic flux, and therefore, this operational mode is desirable for production of glycolytic metabolites or products directly derived from them [137,148,149].

### 5.2. Phosphorus

Phosphorus occurs in cells as phosphorylated organic compounds such as general sugar-phosphates, phospholipids, phosphorylated proteins, RNA, DNA, and ATP. The response to a P-deficiency is typically mediated by a two-component signal transduction system. The sensor kinase component, PhoR, phosphorylates a response regulator that amplifies its own response, increases expression of proteins which scavenge phosphate such as a high-affinity phosphate transporter [150]. Thus, P-limitation has multiple physiological consequences. For example, P-limitation causes a shift in the structure of the cell wall of Gram-positive microbes such as *B. subtilis* from P-containing teichoic acid to teichuronic acid which lacks phosphorus [151,152]. As a result, P-limited *B. subtilis* contain less than half as much cellular phosphate as bacteria grown in excess P, and phages which bind to teichoic acid bind less effectively under P-limited conditions [153,154]. In this case, phosphorylated PhoPR represses *tagAB* operon to restrict teichoic acid synthesis and activates the *tua* operon to stimulate teichuronic acid synthesis [150]. P-limitation triggers the synthesis of phosphate-mobilizing hydrolases such as alkaline phosphatases and ribonucleases [155]. In *S. cerevisiae*, genes responsible for uptake of inorganic phosphates and inositol phosphates are upregulated under steady-state P-limitation at 0.1 h^−1^, and surprisingly, polyphosphates accumulate in this yeast’s vacuoles [141]. Cells appear to use ribosomes for protein synthesis at higher efficiency under phosphate limitation [156], and the RNA content is 6–8-fold greater than expected to be necessary for maintenance of the growth rate, though this RNA is immediately usable when P-limitation is relieved [157].

Isotopic labeling and MFA analysis have been used to compared P-, N-, and C-limited steady-state growth of *B. subtilis* at 0.1 h^−1^ and 0.4 h^−1^ [77], showing that the TCA cycle is severely restricted under P-limitation (only 14% of the flux compared to C-limited conditions, and 12% of flux of N-limited conditions). The rate of glucose uptake is over 30% greater under P-limited conditions compared to C-limited conditions at both growth rates, and the formation of acetate, diacetyl, and acetoin is much greater with those by-products accounting for over one-third of the carbon utilization. A high conversion of malate to pyruvate via malic enzyme and ‘reverse’ flux from oxaloacetate to malate occur at high steady-state growth rate (0.4 h^−1^). Moreover, compared to C-, or N-limited condition, P-limited conditions lead to the greatest partitioning of flux into the pentose phosphate pathway (59% of glucose-6P entered this pathway at 0.1 h^−1^ and 44% at 0.4 h^−1^). High transhydrogenase fluxes are needed to balance the excess reducing equivalents NADPH and NADH [77]. Cells under P-limited conditions also have a greater protein content compared to cells experiencing N-limited or C-limited conditions. Greater CO_2_ production has been widely observed in cells grown under P-limited conditions compared to C-limited conditions, which is attributed to the realignment of metabolic fluxes between the pentose phosphate pathway and TCA cycle [77,141,148,158,159]. P-limited *S. cerevisiae* cultures showed much lower intracellular concentrations of CoA than other nutrient-limited conditions [117].

Specific glucose uptake rate is about 2× greater and acetate formation 20× greater in *E. coli* under P-limited conditions compared to C-limited conditions at 0.2 h^−1^ [160]. P-limited chemostat cultures of *Klebsiella aerogenes* using glucose as the sole carbon source (0.17 h^−1^) secrete polysaccharides, 2-ketogluconate, and gluconate instead of pyruvate [161].

In P-limited *B. subtilis* cultures growing at comparatively high steady-state growth rates (0.4 h^−1^), the futile cycle pyruvate–oxaloacetate–malate (pyruvate carboxylase, malate dehydrogenase, malic enzyme) is induced leading to the net loss of one ATP per cycle [77]. In comparison, in glucose-limited *E. coli* cultures, malic enzyme flux apparently does not occur [116]. Interestingly, malate-to-pyruvate conversion increases with growth rate in *Bacillus megaterium*. However, this reaction does not take place in glucose- and N-limited *B. subtilis* chemostat cultures and is replaced with conversion of oxaloacetate to PEP [77].

In general, the concentrations of nitrogenous bases and nucleosides are elevated under P-limitation compared to N-limitation, and the concentration of these metabolites increases with increasing dilution rate [104]. Because ATP concentration correlates with dilution rate during P-limited growth, ATP availability is thought to control the growth rate under P-limitation [104]. The adenylate energy charge of cells is low, and the ADP concentration relatively elevated, under P-limitation [162].

The formation of several biochemical products has been examined under P-limited conditions. P-limited conditions lead to a 3-fold greater vancomycin production by *Amycolatopsis orientalis* compared to glucose-limited conditions [163], and also increased formation of streptomycin by *Streptomyces griseus* [164] and oxytetracycline by *Streptomyes rimosus* [165]. P-starvation (referred to as limitation) causes the greatest actinorhodin formation in *Streptomyces lividans* [142], and was more effective than N-starvation in generating rhamnolipids by *Pseudomonas aeruginosa* [166]. P-limited conditions lead to the greatest fatty acid formation in *E. coli* compared to C- or N-limited conditions in batch or chemostat culture [167], which was increased further by knockouts in genes associated with flagella [168], a significant energy drain. P-limited cultures generate the greatest yield of glucose from xylose in a strain unable to metabolize glucose and blocked carbon entry into the oxidative pentose phosphate pathway (Δ*zwf*), likely because the final step of this conversion includes the dephosphorylation of glucose-6P [169].

P-limited recombinant *E. coli* cultures showed consistent specific production rate of phenylalanine throughout the fermentation [61]. A hyperproducing *E. coli* mutant showed 8.7 g/L phenylalanine with a 0.44 g/L·h productivity in a P-limited chemostat (0.05 h^−1^) [170]. Compared to S-, Mg-, or K-limitation, P-limitation is the most efficient limiting nutrient for phenylalanine generation in recombinant *E. coli* continuous cultures at 0.1 h^−1^ [171]. Decreased dilution rate (0.03 h^−1^) leads to 16.4 g/L phenylalanine with a productivity of 0.49 g/L·h [171]. Compared to C- or N-limitation, P-limitation resulted in the greatest 3-hydroxypropionate production in engineered strain of *S. cerevisiae* [43]. Because nitrogen is a component of ε-poly-L-lysine, P-limited cultures are used to generate maximal formation using *Streptomyces albulus* under steady-state conditions, and using glucose and glycerol as dual carbon sources attained over 20 mg/g·h specific productivity compared to less than 8 mg/g·h for either single carbon source alone, a result which correlated with much greater activity in aspartate kinase and several other associated enzymes [172]. P-limited conditions increase carbon flux into the isoprenoid pathway in *S. cerevisiae* associated with the upregulation of *PDC6* [173].

Shikimic acid yield was 2.4× greater in *E. coli* grown under P-limited steady-state conditions compared to C-limited conditions, with much fewer by-products under P-limited growth [159,174]. Stipitatic acid was produced to a greater extent by *Penicillium stipitatum* under P-limited conditions compared with N-limited or C-limited conditions, a result which was attributed to the inhibitory effect of phosphate on the polyketide synthesis pathway [82]. Xylitol can be generated from xylose under oxygen-sufficient, P-limited conditions using the yeast *Debaryomyces hansenii* [175].

In *C. necator*, phosphate deficiency discourages the decarboxylation of propionyl-CoA to acetyl-CoA, and consequently leads to a higher fraction of hydroxyvalerate-compared to hydroxybutyrate-containing polyhydroxyalkanoates [176]. P-limitation to sustain growth increases productivity [176]. P-starvation has been proposed for high polyhydroxybutyrate formation because of the lower toxicity of *C. necator* to NH_4_OH compared to NaOH [177].

Several clostridia have been studied for solvent production under P-limited conditions. Under P-limited conditions at the low growth rate of 0.03 h^−1^, *C. acetobutylicum* generates predominantly acetate and butyrate at a pH of 6.0 (more than 90% of products, mole basis), but butanol and acetone at pH 4.3 (87% of total) [178]. This result correlates with changes in observed activities of the enzymes associated pathways, in particular decreased activity in acetate and butyrate-forming enzymes at low pH. Under P-limited conditions at all pH values examined, *Clostridium pasteurianum* accumulates exclusively acetate and butyrate from glucose but ethanol, butanol, and 1,3-propanediol from glycerol [179]. With *Clostridium butyricum*, the greatest H_2_ production is found under P-limited conditions [180]. An extended P-limited process of a *C. acetobutylicm* engineered for butanol production continuously generated 10 g/L butanol at a stable 14 g/L·h productivity and 0.15 g/g yield [181]. A strain with deletions in the butyrate pathway generated 32-fold greater butanol and the unusual products 2-hydroxy-valerate and 2-keto-valerate under P-limited conditions [182].

*E. coli* has a P yield coefficient of 36 g/g [78], while an *E. coli* arginine auxotroph shows a yield coefficient (Y_X/P_) of 34 g/g (Table 1) [79]. The P yield coefficient of *Pseudomonas* C is 28 g/g [83].

Because many carbohydrate metabolites and ATP are phosphorylated, P-limitation likely causes cells to conserve glycolytic metabolites, phosphate ion, and ATP. P-starvation indeed quickly lowers cell adenylate energy charge [183]. We expect P-limited conditions would be uniquely impactful for biochemicals utilizing ATP or having a dephosphorylation near the final step of the production pathway.

### 5.3. Sulfur

Sulfur is present in the amino acids methionine and cysteine, and important metabolites such as S-adenosylmethionine, Coenzyme A (CoA), and lipoic acid. In most media, S is supplied as the sulfate ion, and thus, S-limitation is often studied specifically as sulfate-limitation.

Unsurprisingly, S-limited growth leads to increased transcription of proteins encoding for the uptake of sulfur, such as in *S. cerevisiae* [141], *E. coli* [184]. Under S-limitation or S-starvation, several organisms preferentially express proteins having a low sulfur content [10,141,185,186]. For example, under S-limitation, *S. cerevisiae* upregulates by as great as 50-fold the PDC6 transcript expressing a protein with only 6 sulfur-containing amino acids compared to isozymes PDC1 and PDC5 having 17–18 sulfur-containing amino acids [141], while *K. aerogenes* maintains lower protein content in the cell wall under S-limitation compared to other nutrient-limited conditions, and this remaining protein contains a low sulfur content [187]. The ABC transporter sulfate-binding proteins of *Salmonella typhimurium* and *E. coli* responsible for sulfate uptake in a low S environment themselves contain no sulfur [188]. When *Pseudomonas putida* encounters S-depletion, it replaces proteins having high S content with proteins having lower amounts of cysteine and methionine [86]. *K. aerogenes* also excretes proteins lacking S when grown under S-limited conditions on glucose [161]. This phenomenon has been observed in many microbes, including cyanobacteria [186]. In transitioning between S-limitation and S-enrichment, cells must synthesize new RNA, whereas under C-limitation, the cells use previously synthesized translation machinery, including inactive ribosomes [156,189].

In *E. coli*, S-starvation resulted in a 2.8-fold greater glucose uptake rate than N-starvation, and 40% greater glucose uptake rate than P-starvation, but about 70% less than the glucose uptake rate observed during Mg-starvation [190]. In *B. subtilis*, S-starvation resulted in identical glucose uptake rates as observed during N-, P-starved conditions [190]. S-limitation in *E. coli* led to secretion of pyruvate (yield of 0.33 g/g), succinate (0.11 g/g), and acetate (0.10 g/g) despite the aerobic conditions [190]. S-limited chemostat cultures of *K. aerogenes* also secreted more pyruvate than N-, P- or C-limited growth on glucose [161], while S-limited chemostat cultures of *K. aerogenes* (formerly *Aerobacter aerogenes*) at high growth rate (0.42 h^−1^) showed pyruvate and 2-oxoglutarate accumulation [191]. These observations may be explained by comparing a typical protein (~3% S content by mass) to the sulfur-containing cofactors CoA-SH (4.2% S), lipoic acid (31.1% S), and thiamine pyrophosphate (7.5% S). Each one of these cofactors is a component of the subsequent pyruvate/2-oxoglutarate dehydrogenase step, and the limitation of these cofactors would likely limit metabolic conversion of pyruvate under S-limited conditions [191]. Further evidence identifies lipoic acid as the predominant limiting factor [192]. Interestingly, in contrast to growth on glucose which generated pyruvate and acetate, acetate was the only significant product when *K. aerogenes* was grown on glycerol, mannitol or lactate under S-limited conditions [161].

S-limitation appears to favor plasmid stability compared to C-, N- or P-limitation, particularly at high growth rate [193]. This observation was attributed to the fact that among these four nutrients, only S is not a constituent of nucleic acids. S-starvation resulted in the greatest mevalonate yield from glucose (0.6 mol/mol) compared to Mg-, P- or N-starvation [194,195]. S-starvation (referred to as MgSO_4_ limitation) increased limonene formation by *E. coli* [196]. Similarly, lipid formation elevated when the oleaginous yeast *Rhodosporidium toruloides* became starved for S [197]. The fluxes through the TCA cycle and toward acetate formation were suppressed by S-starvation, and the pentose phosphate pathway appears to be the principal route for NADPH generation [194].

The majority of ribosomal RNA synthesized during S-limitation is believed not to become functional for protein synthesis even after enrichment suggesting that S-limited cultures have limited reserved biosynthetic capability [189]. Hydrogen photoproduction can be prolonged in the green alga *Chlamydomonas reinhardtii* by sulfate starvation [198,199]. Sulfate starvation inactivates photosystem II, resulting in the cells consuming available O_2_, experiencing anaerobic conditions, and inducing hydrogenases which generate H_2_ and sustain the electron transport process [200,201]. Cell viability of microalgae *Chlorella* is more sensitive to nitrogen and phosphate starvation compared to sulfur for production of starch on a large scale [202]. S-starvation leads to the largest carbohydrate content (46.8%) in microalgae *Choleralla sorokiniana* compared to control (2.6%) and is preferred over N- or P-limitation for starch production from algae [203].

A sulfur yield Y_X/S_ of 243 g/g has been reported for *P. putida* after 80 min of S-starvation (Table 1, [86]). In studies using chemostats with different limiting nutrients, the S yield coefficient of *E. coli* cells under sulfur limitation (Y_X/S_) was 278 g/g at a dilution rate of 0.45 h^−1^ [78], and 163 g/g with an arginine auxotroph at 0.4 h^−1^ [79]. 

### 5.4. Magnesium

Magnesium is an integral component of ribosomes [204], and stabilizes the outer membrane in Gram-negative prokaryotes through the creation of ionic bridges [205]. The element as Mg^2+^ is also involved in DNA stability and repair [206], and it plays an important role as an enzyme cofactor. Many complex medium formulations supplemented with carbon source (such as “Lysogeny Broth” medium) become starved for Mg, and can lead to protein acetylation at lysine residues [207]. There have been a few reports on cellular Mg composition (Table 1), and the yield coefficient is highly dependent on cell growth rate. For example, under Mg-limited conditions, *K. aerogenes* showed a yield coefficient of 347–832 g/g (Y_X/Mg_), with the lowest yields occurring at the highest dilution rates [88], while *E. coli* showed a Y_X/Mg_ of 278 g/g at a dilution rate of 0.4 h^−1^, although cell lysis was reported [79]. Similarly, for *B. subtilis* under Mg-limited conditions, the yield coefficient was 714 g/g at a dilution rate of 0.1 h^−1^, and 390 g/g at a dilution rate of 0.6 h^−1^ [87]. *S. cerevisiae* also showed a much greater Mg requirement at higher growth rates in chemostat [208].

Mg-starvation causes *E. coli* cells to restructure the outer membrane and redistribute Mg [209,210]. Although the yield coefficient decreased with increasing dilution rate, Mg-limited *K. aerogenes* cultures do not synthesize intracellular polysaccharides, which is attributed to an impaired ability to synthesize these materials when Mg is limiting [88]. Mg-limitation results in larger and filamentous *E. coli* cells [211,212]. Within 400 steady-state Mg-limited generations, mutations arise in genes involved in the cell-membrane in *E. coli* [209]. Under Mg-limited conditions, both yeast [213] and bacteria [209] reduce the surface hydrophobicity of the lipopolysaccharide (LPS) component of the outer membrane, likely by increasing the proportion of polar sugar residues in the LPS, such that lower amounts of Mg are needed for stabilization of the LPS. Consequently, *S. cerevisiae* shows reduced ability to flocculate under Mg-limitation [213]. When Gram-negative and Gram-positive bacteria were grown together under Mg-limited conditions, Gram-negative bacteria invariably overtook the culture, suggesting that Gram-negative bacteria have a more efficient Mg uptake process, or in other words, a lower value for the saturation constant, *K_S_* [214]. Interestingly, *B. subtilis* exhibits very low incidence of sporulation under P- or Mg-limited conditions compared to carbon- or N-limited conditions [215]. During hyperosmotic stress in *B. subtilis*, cells respond by exporting Mg^2+^ ions to import the K^+^ ion. However, the decrease in free intracellular Mg^2+^ influences energy intensive processes including translation that requires magnesium as a cofactor [216].

There is some research on the effect of Mg-starvation on product formation, and this work is usually conducted in batch studies using a medium in which Mg is depleted prior to the depletion of other nutrients (i.e., starvation). No research was found on the effect of Mg-limitation on product formation. In peptide based medium, Mg-starvation leads to accumulation of acetyl-CoA which is converted to acetyl-phosphate to regenerate CoA for the cellular metabolism [207]. Mg-starvation has been shown to improve butanol formation by *C. acetobutylicum* [217], particularly in the presence of excess zinc. Similarly, a lowered Mg concentration in the second phase of a two-phase fed-batch process increased final butanol titer by 25% using *C. acetobutylicum* [218], a result attributed to ATP demand. In xanthan-producing *Xanthomonas campestris*, restriction of Mg in the medium decreases polysaccharide formation [219], a result attributed to low activity of phosphomannose isomerase. Resuspending a centrifuged culture into a medium lacking Mg, compared to other nutrients, led to the greatest glucose uptake rate and production rates of 3-hydroxypropionate and the flavonoid naringenin [220], thought to be due to a high intracellular PEP concentration and lowered flux through Mg-requiring pyruvate kinase. Mg-depletion during growth also elevated tyrosine and mevalonate formation [195], though compared to S-starvation, Mg-starvation showed lower mevalonate, which was attributed to elevated acetate formation and low NADPH formation because of reduced flux through the pentose phosphate pathway [194]. In comparing *E. coli* cultures from which noncarbon nutrients were depleted before glucose, Mg-starvation showed the greatest glucose consumption rate [190]. This very high glucose consumption rate was accompanied by substantial pyruvate formation (0.74 g/g yield) and an increased PEP pool, effects which were attributed to inactivation of pyruvate dehydrogenase when Mg was depleted [190]. These results could not be explained solely by energy demand, unless the lack of Mg inhibits ATP synthase or increases membrane fluidity. Given that Mg limitation affects membrane structure, it is intriguing to speculate how Mg-limitation impacts not only oxidative phosphorylation, but also the cell’s ability to transport certain products.

Considering the central role that magnesium plays in the cell membrane and in enzymatic conversions involving ATP, the lack of significant research on Mg-limited process for the accumulation of biochemicals is surprising. We envision metabolic circuits involving magnesium which leverage the unique role this ion plays in metabolism.

### 5.5. Iron

Iron is a common element which exists in two cationic oxidation states, and the element often plays a central role in cellular redox processes, and is also critical in strategies pathogens use to infect their hosts [221,222]. Under aerobic conditions and neutral pH, the ferric ion (Fe^3+^) is the principal natural form of iron, a species with low aqueous solubility and the potential to promote the formation of reactive oxygen species. Fe-responsive regulation is often mediated by the Fe-dependent transcriptional factor called Fur. Under conditions of Fe-starvation or Fe-limitation and to ameliorate low Fe biological availability, Fur-based repression is typically relieved, resulting in the expression of numerous genes involved in secreting Fe-chelators, siderophores, and high-affinity siderophore outer-membrane receptors [71,223,224,225]. Fur also regulates the small RNAs which repress genes under Fe-limited conditions [226]. Fur regulated aerobic ribonucleotide reductases encoded by *nrdE* and *nrdF* are manganese-dependent and become active under Fe-starvation [227,228]. Many bacteria also possess an Fe^2+^ transport system, Feo, which is induced and important for survival under anaerobic conditions in which Fe^2+^ is stable [229,230].

Consistent with the theme of cells conserving limited resources, Fe-limitation causes cells to reduce their reliance on pathways which contain significant Fe, such as the tricarboxylic acid cycle (aconitase, fumarate hydratase, succinate dehydrogenase) and the proton-pumping components of the electron transport chain [71,231,232]. This realignment of metabolism has physiological consequences. For example, under Fe-limiting aerobic conditions, *E. coli* generates acetate at a yield of 0.25 g/g at a dilution rate of 0.4 h^−1^, but accumulates predominantly lactate (yield of 0.60 g/g) at 0.1 h^−1^ [71]. Elevated lactate under the most severe steady-state Fe-limitation provides cells a means to oxidize NADH when the Fe-requiring Nuo complex is curtailed, while an increased glucose uptake rate serves to meet ATP demand [71]. Lactate formation is also observed for *Staphylococcus aureus* exposed to Fe-starvation [233]. The reduction in activity of TCA cycle enzymes and accumulation of NADH encourages the formation of ethyl acetate by *Kluyveromyces marxianus* [234] and *Candida utilis* [235]. Because of the restricted capacity of the electron transport chain subject to Fe-limited conditions, cells essentially behave like they are encountering anaerobic conditions. For example, *E. coli* accumulates acetate and formate under Fe-limited steady-state conditions, attains a 60% greater glycolytic flux, and reduces by a factor of 5 the fraction of glucose entering the TCA cycle [236]. More generally, Fe-limitation impacts oxygenation and redox state because numerous electron-carrying enzymes contain Fe-S clusters, and many Fe-containing enzymes or pathways are constrained under Fe-limitation. For example, the nitrogenase enzyme system contains significant Fe [237], and Fe-limitation thus severely reduces nitrogen fixation by *Azotobacter vinelandii* [238]. Notably, lactic acid bacteria, which lack cytochrome and show generally high tolerance to peroxide, do not require iron for growth at all [239,240].

Iron strongly affects anaerobes, which have many Fe-containing enzymes. Under Fe-limited conditions, *C. acetobutylicum* favors from glucose the formation of butanol over acetone at low pH, but the formation of lactate at a pH greater than 5.5 [241], presumably because of the Fe-containing enzymes associated with the conversion of pyruvate to acetyl-CoA (pyruvate:ferredoxin oxidoreductase) and acetoacetate to acetone (acetoacetate decarboxylase). In addition, Fe-limitation leads to a significant reduction in hydrogenase activity [242]. Similarly, for *C. pasteurianum*, Fe-limited conditions favor lactate generation from glucose but 1,3-propanediol from glycerol [179]. The transition between butanol (Fe-excess) and 1,3-propanediol (Fe-limited) from glycerol is attributed to the coupling of H_2_-formation to the ferredoxin-dependent butyryl-CoA dehydrogenase [243]. Fe-limitation causes *C. pasteurianum* to substitute ferredoxin with flavodoxin [244], and flavodoxin is often used as an indicator for the lack of Fe availability [245].

One of the more prevalent transition metals found in many cells (as noted above, Y_X/Fe_ of 5000–7000 g/g under Fe-excess conditions [72]), Fe exemplifies a cofactor found in several specific enzymes that impact metabolic fluxes. A yield coefficient of 1690 g/g was reported under Fe-limited conditions [83]. *E. coli* growing at a dilution rate of 0.45 h^−1^ under Fe-limited conditions showed a yield coefficient of 7700 g/g [78].

## 6. Conclusions

Over the last couple decades, research has focused on using metabolic engineering strategies to generate products. These strategies emphasize redirecting fluxes through modification of the metabolic network of reactions, including optimizing native pathways, expressing genes to code for introduced pathways, and statically or dynamically controlling metabolism to redirect carbon to the desired product. Operational strategies have played a secondary role, and are usually limited to repeated batch processes to attain a high titer or introducing microaerobic conditions for biochemically reduced products. However, limiting a specific nutrient can have profound effects on microbial physiology and metabolic pathways. We propose that the growth under nutrient limitation can *leverage* the native or introduced pathways, which should encourage re-examination of operational strategies. For example, fluxes through competing pathways can be modulated by a nutrient required as a cofactor for one of the designed pathways. Thus, synthetic pathways and circuits can be constructed which by design are regulated by a single nutrient limitation. These include (1) substituting native pathways with enzymes requiring metal ions as cofactors, and which could serve as a control valve to partition flux under the limitation of that metal ion, and (2) designing pathways or metabolic processes which are induced by nutrient limitation.

## Figures and Tables

**Table 1:** Reported yield coefficients for elements using various microorganisms. Where necessary, reported data have been converted so that the yield coefficient is defined as the mass of cells on a dry basis generated per mass of that element consumed (g cells/g element). Each element is available in the medium as a salt or other metabolizable species.

Element	Y_X/element_	Microorganism	Growth Conditions	Operational Condition	Reference
	(g/g)				
Nitrogen (N)		*Bacillus caldotenax*	Pulse Shift; Glucose, methionine, biotin (65 °C)	Chemostat:	[76]
	6			D = 1.2 h^−1^	
		*Bacillus subtilis*	P-limited	Chemostat:	[77]
	7.2			D = 0.1 h^−1^	
	7.3			D = 0.4 h^−1^	
		*Bacillus subtilis*	N-limited	Chemostat:	[77]
	8.1			D = 0.1 h^−1^	
	7.7			D = 0.4 h^−1^	
	5	*Escherichia coli* B/r	Pulse Shift	Chemostat:	[78]
				D = 0.45 h^−1^	
		*Escherichia coli*	C-limited;	Chemostat:	[70]
	7.9 ± 0.3		Cell elemental analysis	D = 0.1 h^−1^	
	8.0 ± 0.4			D = 0.2 h^−1^	
	7.8 ± 0.2			D = 0.3 h^−1^	
	7.8 ± 0.1			D = 0.4 h^−1^	
		*Escherichia coli*	Fe-limited;	Chemostat:	[70]
	7.5 ± 0.1		Cell elemental analysis	D = 0.1 h^−1^	
	7.5 ± 0.1			D = 0.2 h^−1^	
	7.7 ± 0.0			D = 0.3 h^−1^	
	7.5 ± 0.0			D = 0.4 h^−1^	
		*Escherichia coli*	N-limited;	Chemostat:	[70]
	9.7 ± 0.3		Cell elemental analysis	D = 0.1 h^−1^	
	9.9 ± 0.3			D = 0.2 h^−1^	
	8.8 ± 0.1			D = 0.3 h^−1^	
	8.9 ± 0.2			D = 0.4 h^−1^	
		*Escherichia coli*	N-limited;	Chemostat:	[79]
	10	arginine auxotroph containing plasmid	Medium contained arginine and ampicillin	D = 0.4 h^−1^	
		*Escherichia coli* C Δ*ldhA* Δ*poxB* Δ*ppsA*	N-limited	Chemostat:	[80]
	8.35		Medium elemental analysis	D = 0.155 h^−1^	
	8.22			D = 0.208 h^−1^	
	8.26			D = 0.283 h^−1^	
	7.9	*Escherichia coli* W, B, and K-12	Not growth limited;	Exponential Growth at 20 °C or 30 °C	[81]
			Cell elemental analysis		
		*Penicillium stipitatum*	N-limited;	Chemostat:	[82]
	11.5		Medium prepared	D = 0.11 h^−1^	
		*Pseudomonas* C	Pulse Shift; Methanol	Chemostat:	[83]
	9.1			D = 0.32 h^−1^	
	8	*Pseudomonas fluorescens*	Not growth limited;	Exponential Growth at 30 °C	[81]
			Cell elemental analysis		
	7	*Pseudomonas putida*	Not growth limited	Fed-batch	[84]
Phosphorus (P)		*Bacillus caldotenax*	Pulse Shift; Glucose, methionine, biotin (65 °C)	Chemostat:	[76]
	28			D = 1.2 h^−1^	
		*Bacillus subtilis*	K-limited;	Chemostat:	[85]
	53		Cell elemental analysis	D = 0.05 h^−1^	
	59			D = 0.10 h^−1^	
	31			D = 0.20 h^−1^	
	29			D = 0.40 h^−1^	
		*Bacillus subtilis*	N-limited	Chemostat:	[77]
	42			D = 0.1 h^−1^	
	38			D = 0.4 h^−1^	
		*Bacillus subtilis*	P-limited;	Chemostat:	[85]
	77		Cell elemental analysis	D = 0.10 h^−1^	
	59			D = 0.20 h^−1^	
	43			D = 0.40 h^−1^	
		*Bacillus subtilis*	P-limited	Chemostat:	[77]
	96			D = 0.1 h^−1^	
	65			D = 0.4 h^−1^	
		*Escherichia coli* B/r	Pulse Shift	Chemostat:	[78]
	36			D = 0.45 h^−1^	
		*Escherichia coli*	P-limited;	Chemostat:	[79]
	34	arginine auxotroph containing plasmid	Medium contained arginine and ampicillin	D = 0.4 h^−1^	
	34.7 49.8	*Escherichia coli* W *Escherichia coli* B	Not growth-limited; Cell elemental analysis	Exponential Growth at 20 °C or 30 °C	[81]
	41.7	*Escherichia coli* K-12			
		*Pseudomonas* C	Pulse Shift; Methanol	Chemostat:	[83]
	28			D = 0.32 h^−1^	
	43	*Pseudomonas fluorescens*	Not growth-limited;	Exponential Growth at 30 °C	[81]
			Cell elemental analysis		
	42	*Pseudomonas putida*	Not growth-limited	Fed-Batch	[84]
Sulfur (S)		*Escherichia coli* B/r	Pulse Shift	Chemostat:	[78]
	278			D = 0.45 h^−1^	
	163	*Escherichia coli*	S-limited;	Chemostat:	[79]
		arginine auxotroph containing plasmid	Medium contained arginine and ampicillin	D = 0.4 h^−1^	
	244	*Pseudomonas putida*	Low S medium allowed to become exhausted	Batch; 80 min after S starvation	[86]
Magnesium (Mg)		*Bacillus caldotenax*	Pulse Shift; Glucose, methionine, biotin (65 °C)	Chemostat:	[76]
	571			D = 1.2 h^−1^	
		*Bacillus subtilis*	K-limited;	Chemostat:	[85]
	770		Cell elemental analysis	D = 0.05 h^−1^	
	670			D = 0.10 h^−1^	
	530			D = 0.20 h^−1^	
	450			D = 0.40 h^−1^	
		*Bacillus subtilis*	Mg-limited;	Chemostat:	[87]
	714		Cell elemental analysis	D = 0.10 h^−1^	
	567			D = 0.21 h^−1^	
	444			D = 0.405 h^−1^	
	397			D = 0.57 h^−1^	
		*Bacillus subtilis*	P-limited	Chemostat:	[85]
	830			D = 0.10 h^−1^	
	670			D = 0.20 h^−1^	
	500			D = 0.40 h^−1^	
		*Escherichia coli* B/r	Pulse Shift	Chemostat:	[78]
	588			D = 0.45 h^−1^	
		*Escherichia coli* arginine auxotroph containing plasmid	Mg-limited;	Chemostat:	[79]
	278		Medium contained arginine and ampicillin	D = 0.4 h^−1^	
		*Klebsiella aerogenes*	Mg-limited;	Chemostat:	[88]
	832			D = 0.10 h^−1^	
	588			D = 0.20 h^−1^	
	460			D = 0.41 h^−1^	
	432			D = 0.43 h^−1^	
	378			D = 0.60 h^−1^	
	347			D = 0.82 h^−1^	
	236	*Pseudomonas putida*	Pulse shift	Fed-Batch	[84]
	128	*Pseudomonas* C	Pulse Shift; Methanol	Chemostat:	[83]
				D = 0.32 h^−1^	
Iron (Fe)	7700	*Escherichia coli* B/r	Pulse shift	Chemostat	[78]
				D = 0.45 h^−1^	
	5600	*Escherichia coli*	Low iron content;	Batch	[72]
			Cell elemental analysis		
		*Escherichia coli*	Iron-limited;	Chemostat:	[71]
	130,000		Cell elemental analysis	D = 0.10 h^−1^	
	150,000			D = 0.20 h^−1^	
	120,000			D = 0.30 h^−1^	
	100,000			D = 0.40 h^−1^	
	43,000	*Kluyveromyces* *marxianus*	Iron-limited	Chemostat:	[89]
				D = 0.15 h^−1^	
	1700	*Pseudomonas* C	Pulse Shift; Methanol	Chemostat:	[83]
				D = 0.32 h^−1^	
